# Outcomes of medical and surgical treatment for intestinal fistulizing Crohn’s disease

**DOI:** 10.1371/journal.pone.0327784

**Published:** 2025-07-17

**Authors:** Pingrun Chen, Yufen She, Liangfang Wang, Yina Li, Yan Zhang

**Affiliations:** 1 Department of Gastroenterology and Hepatology, West China Hospital, Sichuan University, Sichuan, China; 2 Department of Respiratory and Critical Care, West China Hospital, Sichuan University, Sichuan, China; Barking Havering and Redbridge Hospitals NHS Trust: Barking Havering and Redbridge University Hospitals NHS Trust, UNITED KINGDOM OF GREAT BRITAIN AND NORTHERN IRELAND

## Abstract

**Background:**

Intestinal penetrating complications of Crohn’s disease (CD) are challenging issues, but it is difficult to decide between medical treatment and surgery. This study aimed to evaluate the outcomes of medical and surgical therapy in patients with non-perianal fistulizing CD.

**Objective:**

Our study aimed to evaluate and compare the effectiveness of surgery and medical treatment in CD patients with non-perianal fistulas.

**Materials and methods:**

This retrospective study included all CD patients with non-perianal fistulas. We evaluated the outcomes of medical and surgical therapy. Fistula closure was identified with radiological examinations or ultrasound. Cox regression analysis was subsequently performed.

**Results:**

Sixty patients (37 male) were included. 43.3% of the patients received biological agents as the first therapy, whereas 55% required surgery as the initial therapy, and the remaining 1 patient received azathiopurine alone. 71.7% of the patients achieved fistula closure, with a median follow-up of 32 months. Among the patients who received biologics as the initial treatment, 38.5% achieved fistula closure without the need for surgery, and 50% of the patients underwent surgery. Fistula closure was observed in 69.7% of the patients who underwent surgery as the initial treatment. Enteral nutrition before initial treatment was independently associated with fistula closure.

**Conclusion:**

Although surgery occupies a crucial role in the treatment of patients with non-perianal fistulizing CD, biologics are also effective. Enteral nutrition may increase the probability of fistula closure, especially in patients who undergo surgery as the initial therapy.

## Introduction

Crohn’s disease (CD) is a chronic progressive inflammatory disease involving any part of the whole digestive tract [1]. Strictures and fistulas are common complications of CD. A large study enrolled 2002 patients to assess the long-term evolution of the disease behavior of CD revealed that approximately 60% of the patients developed stricturing or penetrating disease after a 20-year follow-up [2]. Another cross-sectional study included 1557 CD patients and revealed that 10.7% of patients had penetrating CD, 16.7% of patients had both stricturing and penetrating CD, and more than half of the patients had stricturing or penetrating CD [3]. Fistulas are usually an indication for surgery, however, with the development of biologics, these drugs may also be used to treat fistulizing CD according to some studies [4–10]. Although many studies have explored medical therapies, especially biologics, for treating fistulizing CD, most of these studies have focused on perianal fistulas, and the efficacy on non-perianal penetrating CD varied between different studies. Barreiro-de Acosta et al. enrolled CD patients complicated with internal fistulizing disease who received at least 1 biologic agent and reported that fistula closure was observed in 24% of patients [11]. Currently, how to treat CD patients with non-perianal fistulas remains uncertain. Therefore, we aimed to evaluate and compare the effectiveness of surgery and medical treatment in CD patients with non-perianal fistulas.

## Materials and methods

This was a retrospective study, in which we searched the electronic medical records system on August 1st, 2022, and enrolled CD patients who were followed at West China Hospital, Sichuan University, from January 2019 to March 2022. The study protocol conforms to the ethical guidelines of the 1975 Declaration of Helsinki (6th revision, 2008). The study was approved by the hospital ethics committee. (NO:2022–412) The informed consent was not required due to several reasons. Firstly, the data were extracted from the database of the hospital and the data were anonymous. Secondly, this was a non-interventional study. Thirdly, exemption of the informed consent will not have any adverse events on the patients. Patients with a definite diagnosis of CD with intestinal fistula were included in this study. CD was diagnosed by professional physicians on the basis of clinical symptoms, physical signs, laboratory tests, and endoscopic, radiological, and histological findings according to the guidelines from the European Crohn’s and Colitis Organization (ECCO). The evaluation was based on the Montreal classification [12,13]. Patients with perianal fistulas without intestinal fistulas were excluded. The treatment strategy for intra-abdominal abscesses was determined based on clinical presentation, abscess size, imaging characteristics, and multidisciplinary team discussion. Abscesses smaller than 30 mm in diameter were generally managed with antibiotics alone, as previous studies have shown comparable efficacy between conservative and invasive approaches in this group. For abscesses ≥30 mm, percutaneous or surgical drainage was considered, depending on the location, complexity, and the patient’s clinical status. In particular, abscesses larger than 50 mm or those not amenable to percutaneous access were preferentially treated with surgery, despite the associated higher complication rate.

### Definitions and outcomes

A fistula was identified as an abnormal communication between a segment of the intestinal wall and other organs or segments of the digestive tract. A sinus tract is defined as an abnormal tract which ruptured from the intestinal wall and terminated at a blind pouch. Intestinal fistulas and sinuses were confirmed by imaging examinations, such as magnetic resonance enterography (MRE) and computed tomography (CT), or by intestinal ultrasound, endoscopy or surgery [14–16]. Fistulas were classified based on the structure where they originated and terminated. An internal fistula terminated on the structure in the abdominal cavity, while an external fistula terminated on the skin [17]. Mixed fistulas were defined as the coexistence of both internal and external fistulas. Complex internal fistulas were defined as more than 1 internal fistula in a patient. Fistula closure was defined as closure of the fistula through MRE, CT, or intestinal ultrasound. The initial therapy was defined as the first treatment for CD associated fistula, including medical treatment (mainly biological agents in this study) and surgical operation. The decision of surgery or medical treatment was made from the multidisciplinary team which included gastrointestinal physicians, gastrointestinal surgeons, pathologists, nutritionists, radiologists and ultrasonographers, and the patients also took part in the decision-making process. Under the following situations, we suggest surgery as the first treatment over medical treatment: (1) complicated fistulas (such as patients with both internal and external fistulas); (2) fistulas accompanied by stenosis; (3) acute bowel perforation; and (4) high-position fistulas, such as fistulas between the stomach, duodenum, and colon. Under these situations, we recommend medical treatment first: (1) simple fistulas (such as a single sinus or fistula) and (2) a good response to enteral nutrition. The final decision was a result of shared decision-making with patients. Disease severity was assessed by the Harvey-Bradshaw Index [18]. Enteral nutrition before initial treatment was defined as receiving enteral nutritional liquid orally or via a nasogastric tube for 4–8 weeks before the initial treatment.

The primary outcome was the rate of intestinal fistula closure after different treatments. The secondary outcome was to explore the risk factors that may influence patient prognosis following different treatments.

### Statistical analysis

The Statistical Package for Social Sciences (SPSS) 22.0 for Windows was used for statistical analyses. The normality of the distribution of continuous variables was determined by the Kolmogorov–Smirnov test or Shapiro–Wilk test based on the sample size of the analysis. When the sample size was ≤ 50, we used the Shapiro‒Wilk test. When the sample size was > 50, we used the Kolmogorov‒Smirnov test. Normally distributed continuous data are presented as the mean ± standard deviation, and nonnormally distributed data are presented as the median and 25th–75th percentiles. Independent sample t-tests and Mann–Whitney U tests were used for the comparison of normally and nonnormally distributed continuous data, respectively. The chi-square test was used for the comparison of categorical data. Kaplan‒Meier survival analysis and Cox regression analysis were performed to reveal which factors may significantly influence patient prognosis after different treatments. P < 0.05 was considered statistically significant.

## Results

### Patient characteristics

**Fig 1:**
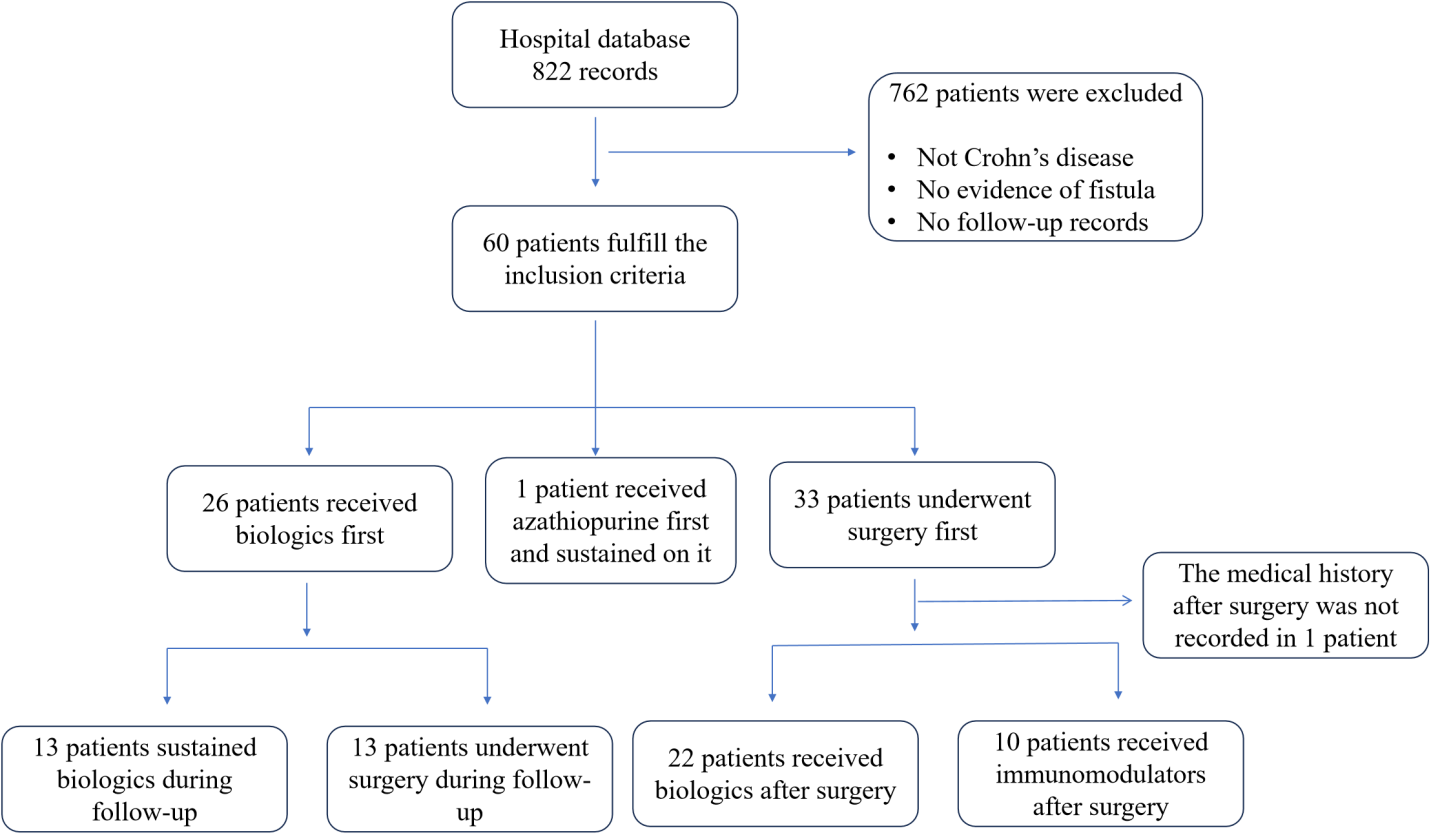
Flowchart of the study.

Sixty patients were ultimately enrolled in this study. Fig 1 showed the flowchart of the study. The patient characteristics are summarized in Table 1. Ileocolonic involvement was reported in 83% of the patients. 42 patients had concomitant stricturing disease behavior at the time of enrollment. 40% of the patients had a history of perianal disease. Disease activities were assessed in 52 patients. Among the 8 patients with mild disease, 3 patients received biologics as first therapy, and the other 5 patients underwent surgery first. 43 patients had moderate disease, 21 of the 43 patients received biologics first, 1 patient was on azathiopurine, and the other 21 patients underwent surgery first. 1 patient had severe disease and underwent surgery first.

**Table 1:** Clinical characteristics of included patients.

Variables	N
Number of included patients (%)	60 (100%)
Male, n (%)	37 (61.7%)
Age (years), median (IQR)	30 (25.25-36.00)
BMI (kg/m2), median (IQR)	17.19 (15.24-18.65)
Smoking status	
Nonsmoking, n (%)	55 (91.7%)
Ex-smokers, n (%)	3 (5%)
Active smokers, n (%)	2 (3.3%)
Extraintestinal manifestation, n (%)	1 (1.7%)
Duration of illness before fistula (years), median (IQR)	3.0 (1.0-8.0)
Montreal classification	
A1, n (%)	2 (3.3%)
A2, n (%)	56 (93.3%)
A3, n (%)	2 (3.3%)
L1, n (%)	4 (6.7%)
L2, n (%)	6 (10%)
L3, n (%)	50 (83.3%)
L4, n (%)	6 (10%)
Disease Activity	
Mild disease, n (%)	8 (13.3%)
Moderate disease, n (%)	43 (71.6%)
Severe disease, n (%)	1 (1.7%)
Accompany with stenosis, n (%)	42 (70%)
Perianal disease, n (%)	24 (40%)
Abscess, n (%)	42 (70%)
Drug history before fistula	
5-ASA, n (%)	27 (45%)
Glucocorticoid, n (%)	17 (28.3%)
Immunomodulators, n (%)	23 (38.3%)
Biologics, n (%)	5 (8.3%)
Medical treatment after fistula	
Immunomodulators in monotherapy, n (%)	11 (18.3%)
Biologics in monotherapy, n (%)	39 (65%)
Biologics combined with immunomodulators, n (%)	9 (15%)

Value of Age, BMI, Duration of illness before fistula is expressed as median (IQR). BMI, body mass index; kg, kilogram; IQR, interquartile range.

42 patients (70%) had a single fistula, while 18 patients (30%) had at least 2 fistulas. Considering the location of the fistula, 50 patients (83%) had internal fistulas or sinuses, 2 patients (3%) had only enterocutaneous fistulas, and 8 patients (13%) had both internal (including 2 patients had sinuses) and external fistulas. Complex internal fistulas were found in 12 patients (20%), and 2 patients had both complex internal and enterocutaneous fistulas (Table 2).

**Table 2:** Characteristics of the fistula.

Type and location of the fistula	N (%)
Type of fistula	
Single fistula	42 (70%)
Mixed fistulas^*^	8 (13.3%)
Complex internal fistulas^#^	12 (20%)
Location of fistula	
Enterocutaneous fistula	10 (16.7%)
Sinus	20 (33.3%)
Rectovaginal fistula	6 (10%)
Enterovesical fistula	2 (3.3%)
Colovesical fistula	1 (1.7%)
Enterocolic fistula	12 (20%)
Enteroenteric fistula	18 (30%)
Cologastric fistula	2 (3.3%)
Coloduodenal fistula	4 (6.7%)
Ileoduodenal fistula	1 (1.7%)

* Mixed fistulas were defined as co-existence of both internal and external fistula.

# Complex internal fistulas was defined as the number of internal fistulae ≥2 in one patient.

2 patients had both mixed fistulas and complex internal fistulas.

### Abscess

42 patients (70%) had concomitant abscesses. The treatments for the abscess included percutaneous drainage, surgical drainage, antibiotics, and total enteral nutrition. 9 patients received percutaneous drainage through ultrasound or CT, 18 patients underwent surgical drainage (3 patients had received both percutaneous drainage and surgical drainage), and 16 patients received medical treatment without drainage (S1 File). Among the 16 patients with medical treatment for abscesses, 6 patients did not have any surgical intervention during follow-up, and they all achieved fistula closure, 10 patients need surgery, and 7 patients (70%) achieved fistula closure. Among other 24 patients who had interventional drainage for treating abscesses, 18 patients (75%) had fistula closure during follow-up.

### Treatment strategy

27 patients had enteral nutrition for 4–8 weeks before initial treatment. Among these patients, 16 patients received biologics as first therapy, 1 patient was on azathiopurine. And 10 patients underwent surgery first.

A total of 46 patients (76.7%) underwent surgery to treat fistulas, and 33 patients (55%) underwent surgery as first-line therapy. Two-thirds of the 33 patients received biologics after surgery, while the others chose immunomodulators. The details of surgery were presented in Table 3. And 10 patients underwent multiple surgeries excluding stoma reversion.

**Table 3:** Treatment strategy of the included patients.

Treatment strategy	N (%)
Patients underwent surgery	46 (76.7%)
The way of surgery	
Laparotomy	36 (60%)
Laparoscopic operation	7 (11.7%)
Surgical procedure	
Ostomy creation and stoma revision (if possible)	30 (50%)
Single surgical procedure	14 (23.3%)
Patients received biologics	48 (80%)
Infliximab	34 (56.7%)
Adalimumab	24 (40%)
Vedolizumab	3 (5%)
Ustekinumab	10 (16.7%)
Switching of biologics	17 (28.3%)

**Fig 2:**
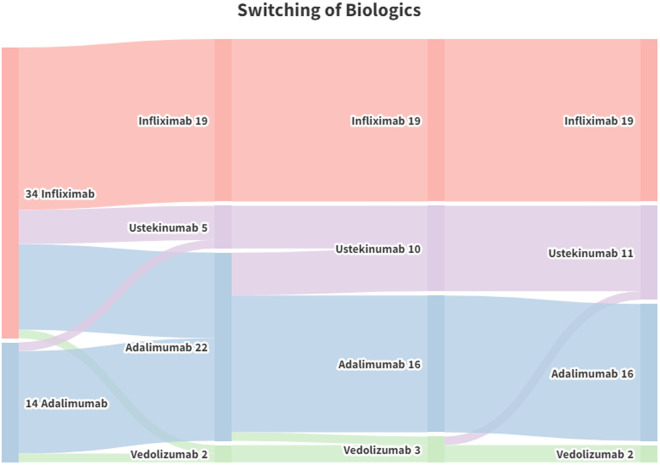
Switching of the biologics. A total of 17 patients experienced switching of biological agents.

48 patients (80%) received biological agents, and 26 patients received biologics as the first choice for treating fistulas (Fig 2). Among these 26 patients, 18 patients chose infliximab, and 8 patients chose adalimumab. Considering the types of biologics used in all patients, anti-TNF-α agents were the most commonly used biologics; 34 patients used infliximab, 24 patients used adalimumab, 10 patients used ustekinumab, and 3 patients used vedolizumab (Table 3). Biologics switching was also recorded. 31 patients received one type of biologic agent without switching, 12 patients received a conversion of two biological agents, 4 patients experienced a conversion of three biological agents, and only 1 patient experienced four types of biologics.

### Need for surgery

Among the patients who chose biological agents as first-line treatment, 50% still needed surgery after a median follow-up of 18.5 months (IQR: 10.0–30.5). 12 patients underwent surgery due to the intestinal fistula itself, 1 patient underwent surgery due to incomplete intestinal obstruction symptoms, and 4 patients experienced abscess recurrence at the time of surgery. 7 patients underwent a single surgical procedure without ostomy formation. The baseline factors that may be associated with the need for surgery were analyzed using univariate Cox regression (S2 File). We found that a history of biologics before fistula (HR = 4.924, 95% CI: 1.263–19.195, P = 0.022) and the number of fistulas ≥ 2 (HR = 4.839, 95% CI: 1.351–17.335, P = 0.015) were significantly associated with the decision to do surgery. However, none of these factors were independently associated with the decision to do surgery.

### Comparison between biologics and surgery strategy

Fistula closure was achieved in 43 patients (71.7%), with a median follow-up of 32 months. Among the 26 patients initially treated with biological agents, 10 (38.5%) achieved fistula closure without surgery, while 9 patients (34.6%) required subsequent surgical intervention to achieve closure. In total, 19 patients (73.1%) from this group experienced successful fistula closure. Among the 33 patients who underwent surgery as the initial treatment, 17 later received biologics and 5 treated with immunomodulators achieved fistula closure. The medical treatment for one patient was not recorded. Overall, 23 patients (69.7%) in this group achieved fistula closure. A comparison of the overall closure rates between the biologics-first and surgery-first groups showed no significant difference (19/26 vs. 23/33, P = 1.0). However, patients who continued with biologics without surgery had a lower fistula healing rate compared to those who underwent surgery (10/26 vs. 23/33, P = 0.02). There was no significant difference in closure rates between patients who underwent surgery after receiving biologics and those who received biologics following surgery (9/13 vs. 17/22, P = 0.698).

**Fig 3:**
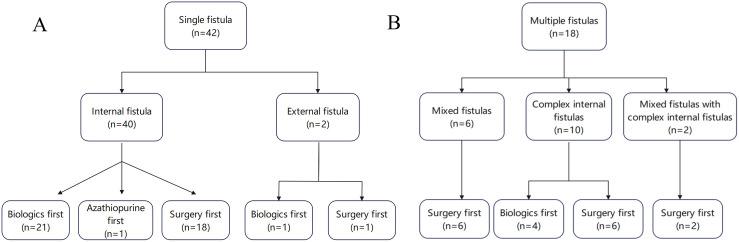
Treatment regarding different types of fistulas (including sinuses). A. Patients with single fistula; B. Patients with multiple fistulas.

We then compared the healing rate regarding the different types of fistulas (Fig 3). For patients with single internal fistula, 21 patients received biologics first, and 16 patients (76.2%) achieved fistula closure, including 10 patients (47.6%) who achieved fistula closure without subsequent surgery. 18 patients underwent surgery initially, and 13 patients (72.2%) achieved fistula closure. We compared the healing rate between patients who received biologics first and patients who underwent surgery first (16/21 vs 13/18, P = 1.0), and between patients who received biologics initially and sustained without surgery and patients who underwent surgery (10/21 vs 13/18, P = 0.192), no significant differences were observed between groups. 2 patients had single external fistula, and 1 patient achieved fistula closure after surgery, while the other patient who received biologics without surgery had persistent fistula. 18 patients had multiple fistulas. Among these patients, only 4 patients with complex internal fistulas received biological agents initially. However, the 4 patients all underwent subsequent surgery and 3 patients (75%) achieved fistula closure. Other patients all underwent surgery first and 9 patients (64.3%) achieved fistula healing.

### Fistula closure

We first analyzed which factors may be significantly associated with fistula closure among patients who chose surgery as initial therapy. Multiple operations (HR = 0.15, 95% CI: 0.034–0.652, P = 0.011) and enteral nutrition before surgery (HR = 3.722, 95% CI: 1.266–10.943, P = 0.017) were of statistical significance after univariant cox regression, and only enteral nutrition before surgery (HR = 2.984, 95% CI: 1.012–8.801, P = 0.048) was independently associated with fistula closure.

**Fig 4:**
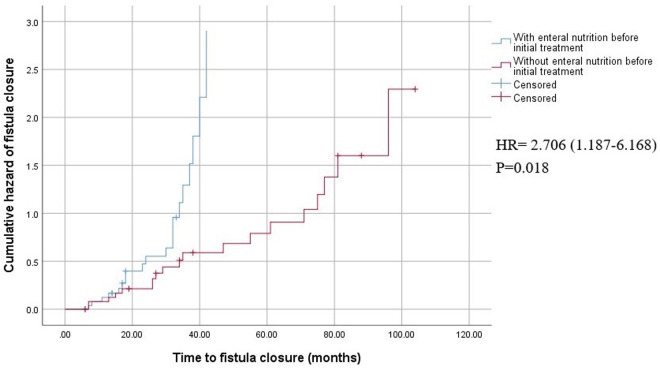
Kaplan-Meier survival curves showing the cumulative hazard of fistula closure with or without enteral nutrition before initial treatment.

Among all 60 patients included, we found that enteral nutrition, initial therapy (biologics or surgery), a shorter duration between fistula discovery and initial therapy, and the use of biological agents during follow-up were associated with fistula closure after univariate analyses (Table 4). Multivariate Cox regression analyses showed that enteral nutrition before initial therapy was independently associated with fistula closure (Fig 4).

**Table 4:** Factors associated with fistula closure.

Variable	Univariate analysis		Multivariate analysis	
	HR (95% CI)	P value	HR (95% CI)	P value
Female	1.025 (0.551-1.909)	0.94		
Age at fistula	1.003 (0.971-1.037)	0.839		
BMI	1.008 (0.893-1.138)	0.898		
Concomitant stenosis	0.936 (0.478-1.831)	0.846		
Accompany with abscess	0.832 (0.415-1.670)	0.605		
Number of fistulae ≥2	0.881 (0.442-1.756)	0.718		
Duration between diagnosis and fistula	1.064 (0.983-1.152)	0.122		
Enteral nutrition before initial treatment	3.026 (1.442-6.350)	0.003	2.706 (1.187-6.168)	0.018
Duration between fistula and initial treatment	0.962 (0.927-0.999)	0.043	0.948 (0.889-1.01)	0.099
History of surgery	1.712 (0.706-4.151)	0.234		
Biologics as initial treatment	2.772 (1.392-5.519)	0.004	1.549 (0.693-3.460)	0.286
Use of biologics during follow-up	2.855 (1.177-6.926)	0.02	1.362 (0.449-4.128)	0.585

HR, hazard ratio; CI: confidence interval; BMI, body mass index.

## Discussion

Our study assessed long-term outcomes in non-perianal fistulizing CD, showing that both surgery and biologics are effective for fistula closure. Enteral nutrition may enhance healing, particularly in patients receiving surgery as initial treatment.

In our study, a total of 43 patients (71.7%) achieved fistula closure after a median follow-up of 32 months. Among patients treated with biologics first, 38.5% achieved fistula closure without surgery. Among the patients who received surgery as first-line therapy, 69.7% achieved fistula closure. For patients with a single internal fistula, there was no significant difference in the healing rate of fistula between patients who received biologics initially and sustained without surgery and patients who underwent surgery first (10/21 vs 13/18, 280 P = 0.192). Among patients with multiple fistulas, 4 patients received biologics first, and all of them underwent subsequent surgery, while the other 14 patients all underwent surgery first and 9 patients (64.3%) achieved fistula healing. Some studies have revealed that biologics could be used for treating intestinal fistulizing CD, but the rate of fistula healing varied among different studies [7,9,11,19]. Anti-TNF agents are classic and effective biologics for treating CD. Kobayashi et al. explored the effectiveness of anti-TNF agents in treating internal fistulas in 93 CD patients. They reported that fistula closure was confirmed in 29% of the patients, and surgery was eventually performed in 44.1% of the patients [7]. Another study included 156 patients who began treatment with an anti-TNF agent for CD with internal fistula and reported that the cumulative probability of fistula healing was 43.9% at 5 years based on imaging analyses, and 43.6% of the patients underwent surgery [9]. Taxonera et al. identified 97 CD patients with entero-urinary fistulas. 33 patients received anti-TNF therapy for treating fistulas, and 45% of these patients achieved sustained remission without surgery [19]. Amiot A and colleagues revealed that anti-TNF agents may be effective in CD patients with enterocutaneous fistulas, with the rate of fistula closure reaching 33% [20]. Another study enrolled 286 patients with enterocutaneous fistulas, and revealed that fistula closure was achieved by means of surgery in 53% of the patients, by medical therapy in 28% of the patients, and through a combination of both in 18% of the patients [10]. Vedolizumab and ustekinumab also have advantages in treating fistulizing CD. Exploratory analyses of data from the GEMINI 2 trial demonstrated that vedolizumab-treated patients had a shorter time to fistula closure and were more likely to have fistula closure at week 52 compared with placebo-treated patients [21]. Another study combined data from the UNITI-1, UNITI-2, and CERTIFI studies reported that complete fistula resolution was attained in 24.7% of all the Ustekinumab treatment groups and 14.1% of the pooled placebo group [22]. Barreiro-de Acosta M et al. carried out a retrospective study with a large population. A total of 760 patients receiving biological agents were included. Fistula closure was observed in 24% of patients, and surgery was performed in 32% of the patients according to their study [11]. Although some patients in our study received more than one biological agent, the initial biologics of all the patients were anti-TNF agents. We found that 38.5% of the patients achieved fistula closure after initial biological treatment without further surgery.

This study found that a history of biologic use before fistula development and having ≥2 fistulas were associated with a higher likelihood of requiring surgery in the biologics-first group. Researchers have also identified several factors that may be associated with subsequent surgery, including longer disease duration, higher Crohn’s disease activity indexes, higher levels of C-reactive protein, lower levels of albumin, abscess, and bowel stricture [7,9,11]. In addition, nonsmokers and combination therapy with immunomodulators seem to be beneficial for reducing the risk of surgery [11]. We did not find factors that were independently associated with surgery. The smaller sample size and shorter duration of follow-up in our study may account for the different results from those of previous studies.

We also found that preoperative enteral nutrition was independently associated with fistula closure for surgery-first patients, while multiple operations may decrease the probability of fistula closure. Although factors such as early initiation of treatment and postoperative biologics appeared beneficial, only preoperative enteral nutrition remained statistically significant in multivariate analysis. The use of biologics during follow-up was associated with a higher probability of fistula healing after univariate analysis. However, these factors were not statistically significant after multivariate Cox regression analysis, which may be due to the small sample size. Postoperative biologics showed a trend toward improved outcomes compared to immunomodulators (77.3% vs 54.5%, HR = 2.624, 95% CI: 0.955–7.214, P = 0.061). According to previous studies, fistula closure is associated with several factors. Baseline characteristics, including a lower number of fistulas, older age, and nonsmokers, were reported to be associated with a higher probability of fistula closure, while factors such as female sex, colonic disease, concomitant stenosis, and lower levels of hemoglobin and albumin may reduce the probability of fistula healing [7,9,11]. Nutritional deficiency is common in CD patients and has a negative effect on surgical outcomes. Many studies have shown that preoperative nutritional supplementation, especially enteral nutrition, can reduce postoperative complications [17,23]. According to the meta-analysis, postoperative complications occurred in 21.9% of patients who received preoperative EN compared with 73.2% of patients who did not receive preoperative EN (OR=0.09, 95% CI: 0.06–0.13, P < 0.001) [23]. The efficacy of enteral nutrition in managing complicated CD has been well established. Yang et al. reported that the number and exudate volume of intestinal fistulae were significantly reduced, and 75% of patients with enterocutaneous fistulas achieved fistula closure after 12 weeks of enteral nutrition [24]. Another study enrolled 42 CD patients with intestinal fistulas, and 20 patients were allocated to receive infliximab and enteral nutrition. After 30 weeks of treatment, 90% of the patients treated with infliximab and enteral nutrition achieved fistula closure [25].

Our study provided a clear and comprehensive comparison of the effectiveness of biological and surgery, demonstrating the efficacy of biologics in treating non-perianal fistulizing CD. Additionally, our findings emphasized the crucial role of enteral nutrition, particularly for patients undergoing surgery. In this study, we summarized the data and proposed treatment recommendations for non-perianal fistulizing CD as a reference for clinical practice, as outlined in the Methods section. Based on our clinical experience, we have established a standardized treatment protocol at our center. Patients will first be given enteral nutrition for about 2 months if possible. For patients with a single internal fistula, we recommend biologics as the initial treatment, while surgery can also be a viable alternative. For patients with complex fistulas, we recommend surgery following enteral nutrition as the primary treatment strategy.

There are several limitations in our study. First, the retrospective design did not allow us to make a decision on treatment strategy at the time of enrollment. Some patients underwent surgery before admission to our hospital. Unlike prospective studies, we cannot perform regular follow-ups with fixed intervals. Second, this was a single-center study conducted in a tertiary hospital in southwestern China, and the patients included cannot represent the whole population. Different living habits, economic statuses, and medical insurance statuses also influence the choice of different therapies. Third, the sample size was small, which may have limited the power of the statistical analysis. Fourth, the suggestions of surgery or medical treatments were influenced by several factors. For instance, patients with single internal fistula were more likely to receive medical treatment first, and this may bring out possible selection bias. Fifth, the recommendations for treating fistulas were not strictly followed. This is a retrospective real-world study, and patients may take other factors into account, including economic burden and post-surgical complications, but not always focus on the efficacy of the treatment. Thus, a larger prospective study is still needed to validate these results.

## Conclusion

In conclusion, this is the first study to report the prognosis of Chinese CD patients with intestinal fistulas. Our study suggested that biological treatment is beneficial in a considerable proportion of CD patients complicated with intestinal fistula and could be an alternative therapy to surgery. Patients who receive adequate enteral nutrition may have better outcomes, especially those who need surgery. Additionally, the use of biologics as initial therapy may be effective in treating single internal fistula. A larger prospective study is urgently needed to validate these results.

## Supporting information

S1 File Flowchart of treatment for abscess. (TIF)

S2 File Factors associated with the decision to do surgery. (DOCX)
