# Supplementary figures and images for: Outcomes of medical and surgical treatment for intestinal fistulizing Crohn’s disease
Source: PLoS One. 2025 Jul 17;20(7):e0327784. doi: 10.1371/journal.pone.0327784 (PMC12270139; doi:10.1371/journal.pone.0327784)

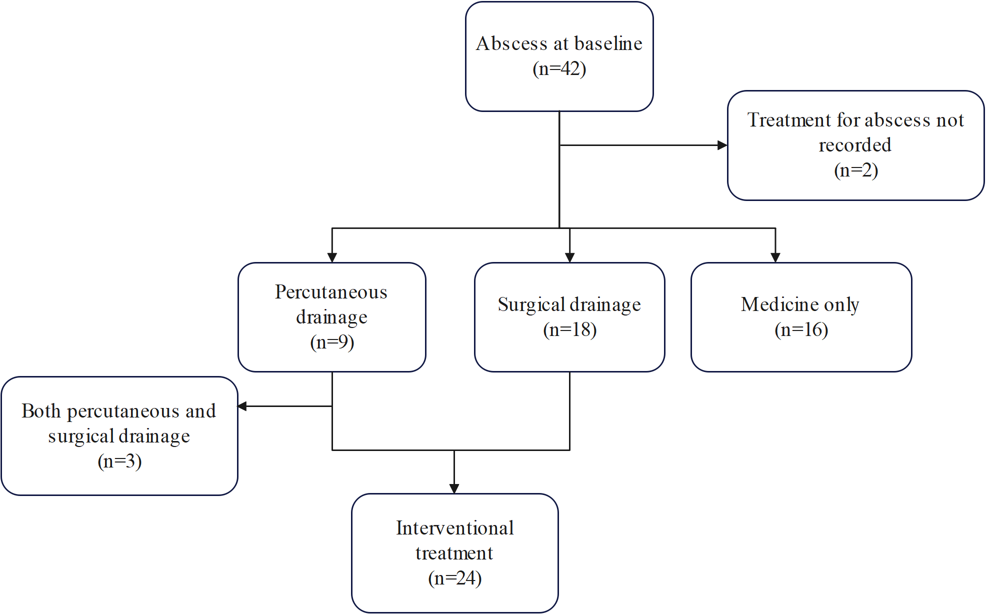

Supplement: S1 File — (TIF) [file pone.0327784.s001.tif]
