# Supplementary material for: Outcomes of medical and surgical treatment for intestinal fistulizing Crohn’s disease
Source: PLoS One. 2025 Jul 17;20(7):e0327784. doi: 10.1371/journal.pone.0327784 (PMC12270139; doi:10.1371/journal.pone.0327784)
Supplement: S2 File — (DOCX) [file pone.0327784.s002.docx]

| Variable | Univariate analysis | | Multivariate analysis | |
| --- | --- | --- | --- | --- |
|  | HR (95% CI) | P value | HR (95% CI) | P value |
| Female | 2.439（0.746-7.972） | 0.14 |  |  |
| Age at fistula | 0.990（0.937-1.046） | 0.712 |  |  |
| BMI | 0.980（0.782-1.228） | 0.860 |  |  |
| Concomitant stenosis | 38.814 (0.231-6513.315) | 0.162 |  |  |
| Abscess at baseline | 0.569 (0.190-1.699) | 0.312 |  |  |
| Number of fistulae ≥2 | 4.839 (1.351-17.335) | 0.015 | 2.058 (0.469-9.029) | 0.339 |
| Disease severity | 0.604 (0.126-2.905) | 0.529 |  |  |
| Duration between diagnosis and fistula | 0.990 (0.867-1.131) | 0.884 |  |  |
| Enteral nutrition before initial treatment | 0.429 (0.144-1.282) | 0.130 | 0.296 (0.080-1.102) | 0.069 |
| Duration between fistula and initial treatment | 0.956 (0.796-1.149) | 0.631 |  |  |
| History of surgery | 0.656 (0.144-2.991) | 0.586 |  |  |
| Ever received biologics before fistula | 4.924 (1.263-19.195) | 0.022 | 5.838 (0.934-36.504) | 0.059 |

Factors associated with the decision to do surgery
